# Efficacy and safety of TAS-115, a novel oral multi-kinase inhibitor, in osteosarcoma: an expansion cohort of a phase I study

**DOI:** 10.1007/s10637-021-01107-4

**Published:** 2021-06-12

**Authors:** Akira Kawai, Norifumi Naka, Akihiko Shimomura, Shunji Takahashi, Shigehisa Kitano, Yoshinori Imura, Kan Yonemori, Fumihiko Nakatani, Shintaro Iwata, Eisuke Kobayashi, Hidetatsu Outani, Hironari Tamiya, Yoichi Naito, Noboru Yamamoto, Toshihiko Doi

**Affiliations:** 1 Department of Musculoskeletal Oncology and Rehabilitation, National Cancer Center Hospital, 5-1-1 Tsukiji, Chuo-ku, Tokyo, 104-0045 Japan; 2 Musculoskeletal Oncology Service, Osaka International Cancer Institute, Osaka, Japan; 3 Department of Breast and Medical Oncology, National Cancer Center Hospital, Tokyo, Japan; 4 Department of Breast and Medical Oncology, National Center for Global Health and Medicine, Tokyo, Japan; 5 Department of Medical Oncology, The Cancer Institute Hospital of JFCR, Tokyo, Japan; 6 Experimental Therapeutics, National Cancer Center Hospital, Tokyo, Japan; 7 Present address: Experimental Therapeutics, National Cancer Center Hospital, Tokyo, Japan; 8 Present address: Division of Cancer Immunotherapy Development, The Cancer Institute Hospital of JFCR, Tokyo, Japan; 9 Department of Orthopaedic Surgery, Osaka University Graduate School of Medicine, Osaka, Japan; 10 Department of Experimental Therapeutics/Breast and Medical Oncology, National Cancer Center Hospital East, Kashiwa, Japan; 11 Department of Gastrointestinal Oncology, National Cancer Center Hospital East, Kashiwa, Japan

**Keywords:** TAS-115, Multi-kinase inhibitor, McDonough feline sarcoma, Met proto-oncogene, Vascular endothelial growth factor receptor, Osteosarcoma

## Abstract

*Background* osteosarcoma is a rare, primary malignant bone tumour with limited available treatments for advanced or recurrent disease, resulting in a poor prognosis for patients. TAS-115 is a novel tyrosine kinase inhibitor under investigation in a phase I study in patients with solid tumours. We report data of osteosarcoma patients in the expansion cohort of this ongoing study. *Patients and methods* an analysis of this multicentre, open-label study was performed 6 months after the final patient was enrolled, and included patients aged ≥15 years, with unresectable or recurrent osteosarcoma, and who had refractory to standard therapy or for whom no standard therapy was available. TAS-115 650 mg/day was orally administered in a 5 days on/2 days off schedule. *Results* a total of 20 patients with osteosarcoma were enrolled. The most common adverse drug reactions (ADRs) were neutrophil count decreased (75%), aspartate aminotransferase increased (50%), and platelet count decreased (50%); 85% of patients had grade ≥ 3 ADRs. Long-term disease control (>1 year) with TAS-115 was achieved in three patients. The best overall response was stable disease (50%); no patient achieved a complete or partial response. Median progression-free survival was 3 months; 4-month and 12-month progression-free rates were 42% and 31%, respectively. *Conclusion* the safety and tolerability of TAS-115 and long-term disease stability for patients with unresectable or recurrent osteosarcoma were confirmed in this study, suggesting that TAS-115 is a promising novel therapy for advanced osteosarcoma patients. Trial registration number: JapicCTI-132333 (registered on November 8, 2013).

**Supplementary Information:**

The online version contains supplementary material available at 10.1007/s10637-021-01107-4.

## Introduction

Osteosarcoma is a rare, primary bone malignancy, with a worldwide annual incidence of 3.4 million [1]. Despite being rare, osteosarcomas are the most common primary bone tumour in children and adolescents; the incidence peaks during the 20s [2]. Current therapy aims to prevent microscopic metastasis and comprises local surgery and neoadjuvant/adjuvant chemotherapy [3], namely, high-dose methotrexate, doxorubicin, and cisplatin (MAP) [4]. However, no effective treatment for osteosarcoma patients after MAP treatment has yet been confirmed.

The 5-year survival rate for patients with advanced osteosarcoma is ~62% [1]. About 20% of patients have metastases at the time of diagnosis [5], with an estimated survival rate of 20% [6]. Bone and pulmonary metastases are prognostic factors of poor survival [2, 5]; pulmonary metastases are more common (80%) [5].

No new drugs have been approved since the 1980s, except for mifamurtide (EU approval in 2009) [7]. In a pooled data analysis, the rate of recurrence-free survival in 96 patients with advanced or recurrent osteosarcoma was 12% at 4 months [8]. In a study in Japanese osteosarcoma patients with pulmonary metastases [9], most patients with inoperable disease died within 1 year. Thus, the unmet need for osteosarcoma treatment is high.

Although various factors (such as vascular endothelial growth factor [VEGF], insulin-like growth factor 1, platelet-derived growth factor [PDGF], human epidermal growth factor receptor 2, and hepatocyte growth factor receptor [MET]) overexpressed in osteosarcoma tumour cells have been suggested as therapeutic targets [10], no targeted agents, including kinase inhibitors, have yet been approved for this condition. This is attributed to the rarity and genetic heterogeneity of osteosarcoma [3]. However, multi-targeted tyrosine kinase inhibitors are reportedly effective in osteosarcoma. A Phase II study of regorafenib (which targets the VEGF receptor [VEGFR], PDGF receptor [PDGFR], fibroblast growth factor receptor, angiopoietin receptor TIE2, and the proto-oncogenes KIT, RET, RAF-1, and BRAF) in adults with metastatic osteosarcoma reported a median progression-free survival (PFS) of 16.4 weeks (95% confidence interval [CI] 8.0–27.3) in the regorafenib group and 4.1 weeks (3.0–5.7) in the placebo group [11]. A Phase II study of cabozantinib (which targets VEGFR-2, the AXL receptor tyrosine kinase, and c-MET) in 42 patients with heavily pretreated, advanced osteosarcoma reported a median PFS of 6.2 months [12].

TAS-115, a novel oral multi-kinase inhibitor, inhibits the autophosphorylation of MET, VEGFR, PDGFR, and Feline McDonough Sarcoma oncogene [13, 14]. In non-clinical osteosarcoma studies, TAS-115 inhibited tumour enlargement and progression of pulmonary metastasis [13], demonstrating its potential antitumour effects at lung metastatic tumour sites. A Phase I study aimed to evaluate TAS-115 treatment for advanced solid tumours is ongoing [15]. Based on the efficacy of TAS-115 among patients with bone lesions in parts 1 and 2 [15], osteosarcoma patients were enrolled in the expansion cohort of the study. Herein, we report the safety and efficacy results of osteosarcoma patients treated with TAS-115 in the expansion cohort.

## Methods

### Study design

The study design was previously reported [15]. Briefly, this multicentre, open-label, dose-titration study centrally enrolled patients with solid tumours, was initiated on December 1, 2013, and is ongoing. Analyses were prespecified (Supplementary Methods). Here, we report the analysis performed 6 months after the final patient was enrolled (data cut-off: August 19, 2018).

The study has three parts: a dose-escalation cohort using a traditional 3 + 3 design (part 1), a dosing schedule investigation cohort (part 2), and an expansion cohort to assess the safety at the maximum tolerated dose (MTD) or lower doses among additional patients (expansion part) (Supplementary Figure 1). In the expansion part, after the MTD was determined, an additional safety and efficacy investigation was performed using a dose ≤MTD based on the development of adverse drug reactions (ADRs) and pharmacokinetic data.

The study protocol was approved by the institutional review board at each participating site and was conducted per the ethical principles of the Declaration of Helsinki, Pharmaceutical Affairs Law, and Good Clinical Practice. All patients provided written informed consent [15].

### Patients

Complete inclusion/exclusion criteria are provided in the Supplementary Methods. Briefly, eligible patients were aged ≥15 years and had osteosarcoma refractory to standard therapy or for which no appropriate standard therapy was available, Eastern Cooperative Oncology Group performance status (ECOG PS) 0 or 1, and adequate bone marrow reserve and renal and liver function at enrolment. Exclusion criteria included serious medical conditions, surgery within 28 days of enrolment, and radiation therapy or other anticancer therapy within 21 days of enrolment.

### Treatment

The treatment regimen comprised TAS-115 650 mg/day, orally administered in a 5 days on/2 days off schedule. If patients met any of the following criteria, TAS-115 treatment was discontinued: neutrophil count <500/mm^3^, platelet count <50,000/mm^3^, grade ≥ 3 nonhaematologic toxicity, and any other toxicity at the investigator’s discretion. Criteria for TAS-115 dose reductions, treatment resumption, precautions, and prohibited concomitant medications and therapies are provided in the Supplementary Methods.

### Procedures and assessments

Examinations included body weight and vital signs, laboratory tests, electrocardiogram, tumour bone markers, bone metabolism markers, and ECOG PS assessment. Imaging tests (e.g., computed tomography [CT], magnetic resonance imaging, bone scintigraphy, positron-emission tomography [PET], and radiographs) were performed before enrolment. Using Day 1 of cycle 1 as baseline, imaging tests were performed on Days 42 and 84, and subsequently, every 84 days. Bone scintigraphy was performed to assess metastasis. BONENAVI® software was used to calculate the bone scan index (BSI).

### Endpoints

Adverse events (AEs) and ADRs were recorded and graded according to the Common Terminology Criteria for Adverse Events (CTCAE version 4.03). Efficacy was based on response evaluated by imaging and antitumour effect assessed according to the Response Evaluation Criteria in Solid Tumors (RECIST version 1.1). Efficacy endpoints were overall response, best overall response, PFS, and disease control rate (DCR). PFS was the period from enrolment to the confirmation date of disease progression or death by any cause before the completion date of the post-observation period, whichever occurred first. DCR was defined as the percentage of patients whose best overall response was complete or partial response (CR/PR) or who continued to have stable disease (SD) for ≥12 weeks. Bone scan response was based on BSI.

### Statistical methods

In this subgroup analysis of the main study population, no statistical rationale was applied to calculate the sample size. The response rate, disease control rate, and corresponding 95% CIs were calculated based on best overall response. PFS was estimated using the Kaplan–Meier method. All statistical processing was performed using SAS Version 9.4.

## Results

### Patients

Of 55 patients enrolled in the expansion cohort from five sites, 20 had osteosarcoma and were included in this analysis. Baseline demographic and clinical characteristics of osteosarcoma patients are shown in Table 1. Patients had a median age of 30 years; 90% did not have a primary lesion. Most patients (90%; *n* = 18) had previously received a regimen equivalent to MAP. Of patients who had received prior therapies other than MAP, 35% (*n* = 7) each had received one and two treatment regimens and 25% (*n* = 5) had received ≥3 regimens. The most common metastasis site was lung (40%; *n* = 8), with 25% (*n* = 5) presenting with both lung and bone metastases.

**Table 1:** Patient baseline demographic and clinical characteristics

**Baseline characteristics**	***N*** **= 20**
Age, years	
Median (range)	30 (16–64)
Sex, *n* (%)	
Female / Male	12 (60) / 8 (40)
ECOG PS, *n* (%)	
0 / 1	13 (65) / 7 (35)
Primary lesion, *n* (%)	
Yes	2 (10%)
No	18 (90%)
Prior treatment regimen, *n* (%)	
Regimen equivalent to MAP	18 (90)
Number of other regimens received	
0	1 (5)
1	7 (35)
2	7 (35)
> 3	5 (25)
Site(s) of metastasis, *n* (%)	
Lung	8 (40)
Lung and bone	5 (25)
Bone	4 (20)
Other (except for lung or bone)	3 (15)

*ECOG PS* Eastern Cooperative Oncology Group Performance Status, *MAP* methotrexate, doxorubicin, and cisplatin

### Safety

The overall incidence of grade ≥ 3 ADRs (≥10%) was 85% (*n* = 17); the most common were neutrophil count decreased, white blood cell count decreased, hypophosphataemia, and anaemia (Table 2). Common ADRs occurring at an incidence >30% were neutrophil count decreased, aspartate aminotransferase increased, platelet count decreased, white blood cell count decreased, face oedema, alanine aminotransferase increased, and hypophosphataemia. Serious AEs are described in the Supplementary Results and Supplementary Table 1.

**Table 2:** Adverse drug reactions of any grade with an incidence of ≥20%

**Preferred term,** ***n*** **(%)**	**G1**	**G2**	**≥G3**	**Total**
Neutrophil count decreased	0	3 (15)	12 (60)	15 (75)
Platelet count decreased	4 (20)	2 (10)	4 (20)	10 (50)
Aspartate aminotransferase increased	7 (35)	1 (5)	2 (10)	10 (50)
White blood cell count decreased	0	2 (10)	7 (35)	9 (45)
Face oedema	7 (35)	2 (10)	0	9 (45)
Alanine aminotransferase increased	4 (20)	3 (15)	1 (5)	8 (40)
Hypophosphataemia	0	2 (10)	5 (25)	7 (35)
Anaemia	0	2 (10)	3 (15)	5 (25)
Rash	2 (10)	2 (10)	1 (5)	5 (25)
Pyrexia	2 (10)	2 (10)	1 (5)	5 (25)
Nausea	4 (20)	1 (5)	0	5 (25)
Diarrhoea	5 (25)	0	0	5 (25)
Blood creatine phosphokinase increased	2 (10)	1 (5)	1 (5)	4 (20)
Lipase increased	2 (10)	2 (10)	0	4 (20)
Amylase increased	3 (15)	0	1 (5)	4 (20)
Fatigue	3 (15)	1 (5)	0	4 (20)
Blood lactate dehydrogenase increased	4 (20)	0	0	4 (20)

*G* Grade

The incidence of serious ADRs was 15% (*n* = 3); these included one case each of enterocolitis, pyrexia, and rash. Eleven (55%) patients required a dose reduction and 12 (60%) patients required a dose interruption due to an ADR (Supplementary Table 2). Of note, one patient (5%) presented a grade 1 pneumothorax. No deaths occurred due to ADRs.

### Efficacy

**Fig. 1:**
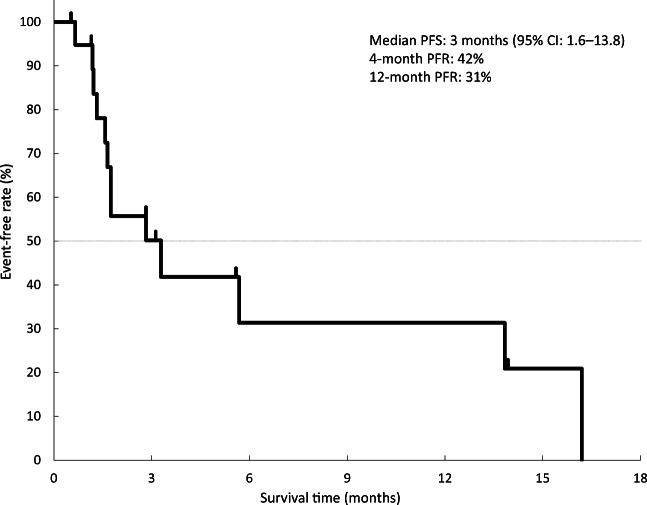
Progression-free survival of patients with osteosarcoma. CI, confidence interval; PFR, progression-free rate; PFS, progression-free survival

The best overall response was SD (50%; *n* = 10); the DCR was 40%. No patient achieved CR/PR. Median PFS was 3 months (95% CI: 1.6–13.8); 4-month and 12-month progression-free rates (PFR) were 42% and 31%, respectively (Fig. 1).

**Fig. 2:**
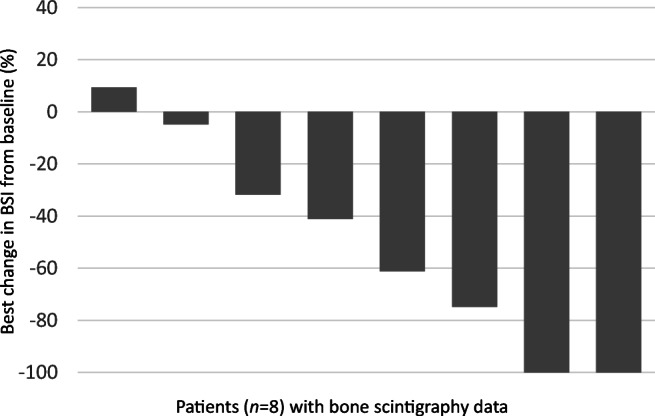
Bone scan response from baseline by BSI (Waterfall plot). BSI, bone scan index

**Fig. 3:**
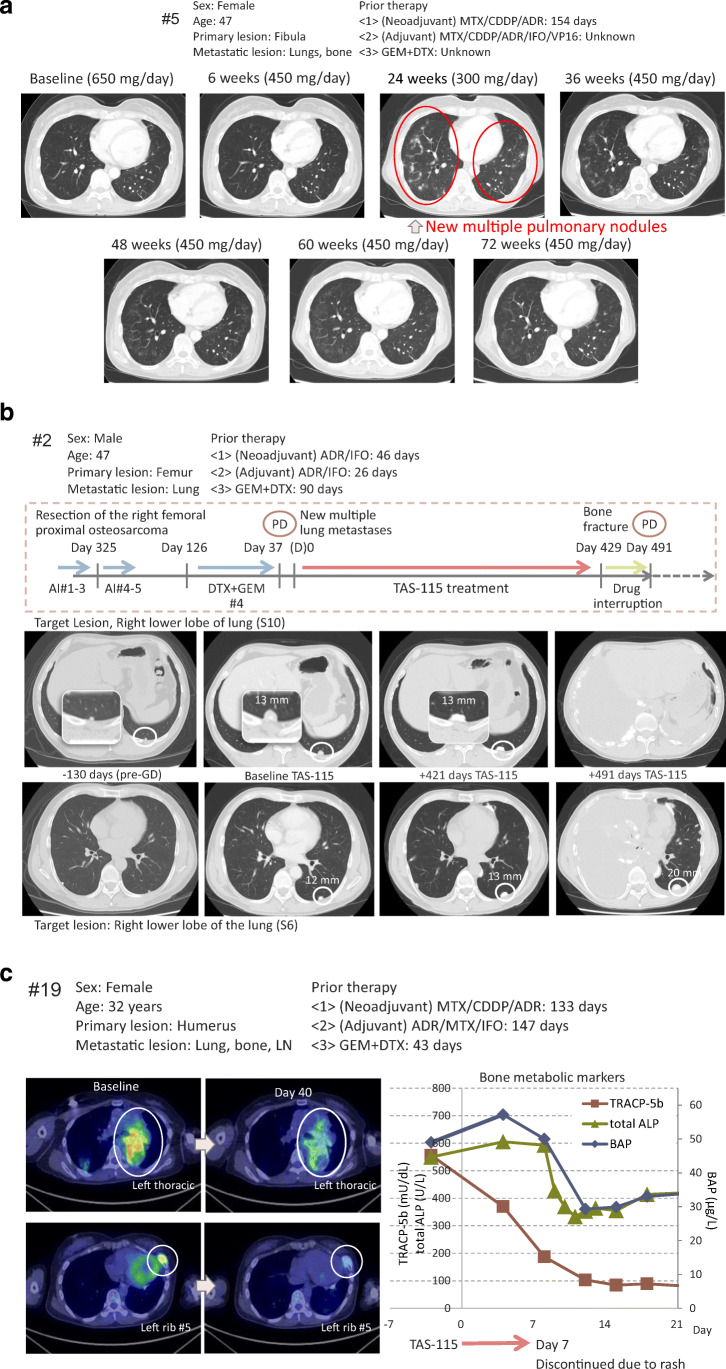
Summary and radiographic images of three individual patient cases. **a** Case #5. Female patient with primary osteosarcoma lesion in the fibula and metastases to the lungs and bone initiated TAS-115 treatment at 650 mg/day. After 3 weeks, the dosing level was reduced to 450 mg/day owing to bone marrow suppression. A further dose reduction to 300 mg/day was decided at 12 weeks owing to neutropenia. Although CT imaging at 24 weeks showed new multiple pulmonary nodules and indicated progressive disease, TAS-115 treatment continued with incremental dosing increases to 450 mg/day. CT imaging after 36 weeks showed shrinkage of the pulmonary nodules. **b** Case #2. Male patient with primary osteosarcoma lesion in the femur and metastases to the lungs started TAS-115 treatment at a dose of 650 mg/day and showed a significant response to treatment by bone scintigraphy. As the patient presented with grade 3 neutrophil count decreased in cycle 1, the dose was decreased to 450 mg/day from cycle 2 onwards. The patient remained in SD for a long period. On Day 429, the patient had a bone fracture secondary to a misstep. TAS-115 administration was stopped owing to bone fracture treatment. On Day 491, the patient discontinued the study because a new neoplasm was observed via CT; pulmonary metastasis was enlarged, and a large amount of pleural fluid was observed. **c** Case #19. Female patient with primary osteosarcoma lesion in the humerus and metastases to the lungs, bones, and lymph nodes showed signal reduction of the left thoracic and left rib metastasis by PET-CT at 6 weeks. However, the patient discontinued treatment due to rash at 1 week. ADR, Adryblastin® (doxorubicin); AI, Adryblastin® (doxorubicin and ifosfamide; ALP, alkaline phosphatase; BAP, bone alkaline phosphatase; CDDP, cisplatin; DTX, docetaxel; GD, gemcitabine and docetaxel, GEM, gemcitabine; IFO, ifosfamide; LN, lymph nodes; MTX, metothrexate; PD, progressive disease; PET-CT, Positron Emission Tomography/Computed Tomography; TRACP, tartrate resistant acid phosphatase; VP-16, etoposide

Of the eight patients with bone scintigraphy data, six (75%) had ≥30% BSI reduction (Fig. 2). Supplementary Figure 2 shows the individual change in bone scan response from baseline in the eight patients who underwent bone scintigraphy and indicates the general trend towards BSI reduction over time. Three individual patient cases are described in Fig. 3.

### Exposure

**Fig. 4:**
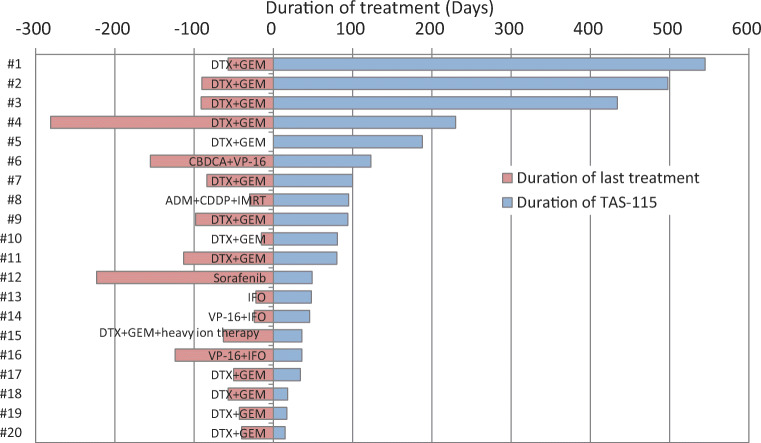
Comparison of treatment duration between previous regimens and TAS-115. ADM, adriamycin; CBDCA, carboplatin; CDDP, cisplatin; DTX, docetaxel; GEM, gemcitabine; IFO, ifosfamide; NE, not evaluable; PD, progressive disease; SD, stable disease; VP-16, etoposide

Figure 4 shows the durations of prior treatments and TAS-115. In nine (45%) patients, TAS-115 treatment duration was longer than previous treatment duration. The median TAS-115 treatment duration was 83 days and the mean (standard deviation) relative dose intensity was 65% (22%). Sixteen patients (80%) discontinued treatment because of disease progression (*n* = 10; 50%), ADR (*n* = 3; 5%), and investigator’s discretion (*n* = 1; 5%) (Supplementary Table 2).

## Discussion

In this expansion cohort of an ongoing Phase I study of TAS-115 in patients with solid tumours [15], the most common grade ≥ 3 ADRs with TAS-115 were neutrophil count decreased, white blood cell count decreased, and hypophosphatemia, which was comparable with parts 1, 2, and the expansion cohort of the overall Phase I study [15]. The incidence of grade ≥ 3 neutropenia and thrombocytopenia related to the study drug in this study was higher than that reported in a previous study of cabozantinib [16]. The higher incidence is possibly because the number of previous treatments in our patients was higher: 90% of patients received a regimen equivalent to MAP. Additionally, 60% of patients received at least two regimens as other treatments. The proportions of patients requiring a dose reduction due to neutropenia and thrombocytopenia were 30% (*n* = 6) and 20% (*n* = 4), respectively, but the duration of these grade ≥ 3 ADRs was about 1 week. The incidence of febrile neutropenia among patients was 5% (*n* = 1). While the incidences of these grade ≥ 3 ADRs were higher compared with the previous study of cabozantinib, these ADRs were manageable.

The incidences of dose reductions and interruptions of TAS-115 treatment due to ADRs were 55% and 60%, respectively. The incidence of discontinuation due to ADRs was 15%; thus, TAS-115 is tolerable with dose reduction or temporary treatment suspension. Long-term disease control (>1 year) of TAS-115 was achieved in three patients; treatment durations with TAS-115 were longer compared with previous therapeutic regimens in around half of patients, suggesting a possible prolonged exposure to TAS-115.

In terms of TAS-115 efficacy, the PFR after 4 months of TAS-115 treatment was 42%; this was a higher percentage than the 4-month PFR of 12% reported for osteosarcoma patients in a pooled analysis of seven Phase II, randomised controlled trials of refractory/recurrent paediatric solid tumours using conventional chemotherapy agents [8]. In a recent Phase II study of regorafenib [11], the PFR at 2 months (8 weeks) was 65%. We consider that the difference in PFR compared with the present study stems from differences in the two patient populations: patients in the regorafenib study were at earlier stages of osteosarcoma and had a shorter treatment history (one or two previous lines of treatment). Conversely, a Phase II study of cabozantinib included heavily pretreated patients, and 33.3% of patients had not progressed by 6 months [12], which is more comparable with our own data.

CR/PR was not observed. In 10 out of 20 (50%) patients, SD lasted for extended periods compared with the previous treatment. A recent study reported that 61% of pulmonary metastases were calcified in osteosarcoma patients [17], and tumour volume shrinkage according to RECIST criteria may not correctly reflect drug efficacy in osteosarcoma.

In this study, we used change in BSI on bone scans as an assessment to evaluate treatment response/benefit. However, we do not consider it feasible to draw conclusions regarding the relationship between BSI, lung CT changes, and efficacy because the number of patients was small and the duration of the assessment was short. To determine whether early changes in BSI can be used as an imaging biomarker for clinical benefit, further investigations are required.

The efficacy of various biologics and small molecules was evaluated in osteosarcoma, yielding far from promising results [18–20] and a lack of improvement in survival rates [2, 7]. However, inhibition of PDGFR, VEGFR, and MET in non-clinical and clinical studies resulted in antiproliferative effects [21–24]. Mifamurtide, a potent activator of immune response, activates macrophages and monocytes and has shown efficacy in combination with MAP therapy [25]. TAS-115 is expected to exert a similar inhibitory effect and may also suppress the differentiation of M2-like macrophages, involved in cancer-related inflammation, tumour growth, and progression [26], via colony-stimulating factor-1 receptor (CSF-1R) inhibition [27], as well as improve antitumour immune defences in the tumour microenvironment. A case analysis (Fig. 3c) indicated that despite TAS-115 treatment discontinuation after 1 week, PET-CT imaging at 6 weeks confirmed signal attenuation of bone metabolic markers, which we hypothesise may have been due to improved antitumour immune defences in the tumour microenvironment by CSF-1R inhibition.

In conclusion, the safety and tolerability profile in this study and the confirmation of long-term disease stabilization, suggest that TAS-115 is a promising treatment for advanced osteosarcoma. The main study limitations were the open-label, non-comparative design and small sample size, so further clinical investigation of TAS-115 should be conducted in these patients.

## Supplementary Information

ESM 1 (DOCX 169 kb)

ESM 2 (DOCX 52.7 kb)

ESM 3 (DOCX 56.5 kb)

## Declarations

### Collaborators

The authors have no collaborators to declare.
