# Supplementary material for: Efficacy and safety of TAS-115, a novel oral multi-kinase inhibitor, in osteosarcoma: an expansion cohort of a phase I study
Source: Invest New Drugs. 2021 Jun 12;39(6):1559–67. doi: 10.1007/s10637-021-01107-4 (PMC8541973; doi:10.1007/s10637-021-01107-4)
Supplement: Supplementary file 1 — (DOCX 169 kb) [file 10637_2021_1107_MOESM1_ESM.docx]

**SUPPLEMENTARY FIGURES**

**Supplementary Figure 1.** Study design


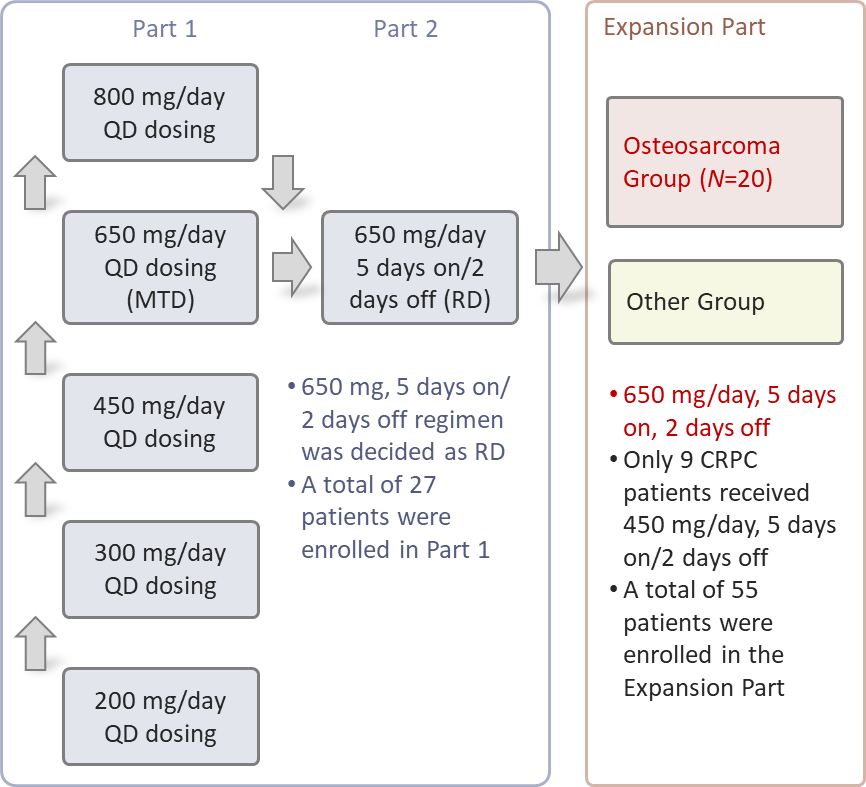


CRPC, castration-resistant prostate cancer; MTD, maximum tolerated dose; RD, recommended dose; QD, once a day

**Supplementary Figure 2.** Percent change in bone scan response (by BSI) from baseline


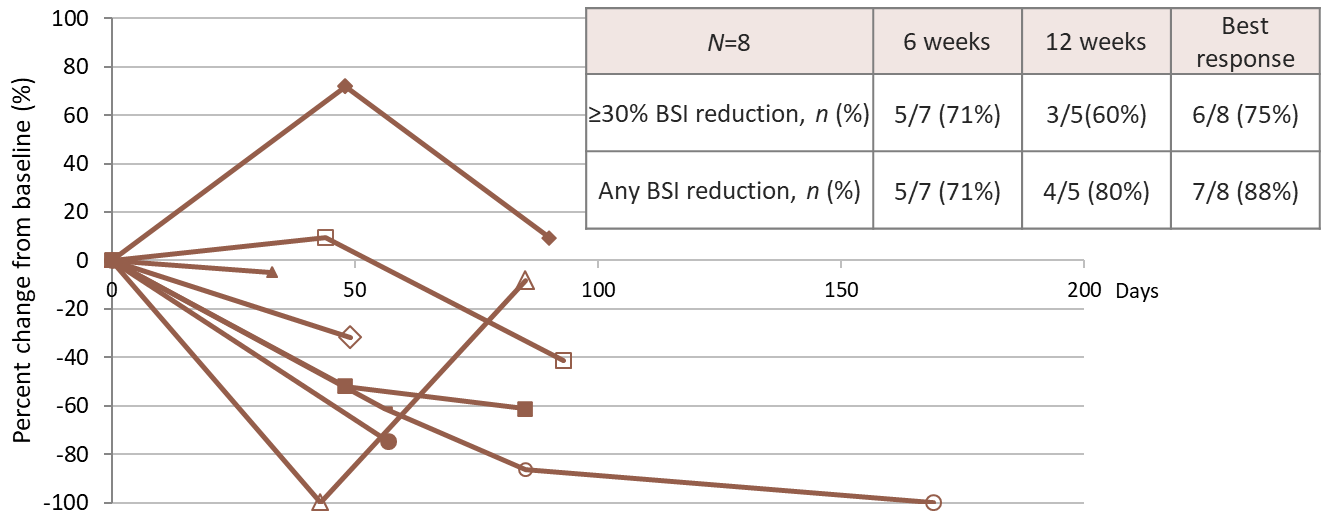


BSI, bone scan index
