# Supplementary material for: Efficacy and safety of TAS-115, a novel oral multi-kinase inhibitor, in osteosarcoma: an expansion cohort of a phase I study
Source: Invest New Drugs. 2021 Jun 12;39(6):1559–67. doi: 10.1007/s10637-021-01107-4 (PMC8541973; doi:10.1007/s10637-021-01107-4)
Supplement: Supplementary file 2 — (DOCX 52.7 kb) [file 10637_2021_1107_MOESM2_ESM.docx]

**SUPPLEMENTARY METHODS**

***Timing of statistical analyses***

It was prespecified in the protocol that statistical analyses would be conducted at three time points: (1) at the end of cycle 1 (primary analysis performed when the last patient to be enrolled completed part 2, and the additional safety investigation performed when the 40^th^ patient completed part 2); (2) 6 months after the enrolment of the last patient; (3) at the end of observation for the last patient.

***Inclusion criteria***

Patients who met the following criteria at the time of enrolment were included in this study:

(1) Patients who gave their written consent to become subjects within 14 days before enrolment in this study; (2) patients who were histologically or cytologically determined to have solid tumours (malignant tumour); (3) patients who did not respond to standard treatment or similar treatment or for whom no appropriate therapy is available; (4) patients determined to have a lesion based on objective documents within 14 days before enrolment in this study (regardless of the presence or absence of the target lesion); (5) patients who could receive oral administration; (6) patients whose age at the time of informed consent was ≥20 years (for patients with malignant bone tumour, ≥15 years); (7) patients who meet the following criteria for bone marrow, liver, and kidney function, based on data within 3 days before enrolment in this study: white blood cell count: ≤10000/mm^3^_,_ neutrophil count: ≥1500/mm^3^, haemoglobin: ≥9.0 g/dL, platelet count: ≥75000/mm^3^, and serum total bilirubin: ≤1.5 mg/dL; creatinine clearance (Ccr) estimate: ≥50 mL/min (Cockcroft-Gault formula); (8) patients whose Eastern Cooperative Oncology Group (ECOG) Performance Status (PS) was 0 to 1; and (9) patients who were expected to survive for 60 days or more after enrolment in this study.

***Exclusion criteria***

Patients who met any of the following criteria at the time of enrolment were excluded from this study: (1) Patients with a history of TAS-115 use; (2) patients with a past history of severe drug allergy; (3) patients with clinically significant electrocardiogram abnormalities or with a clinically significant heart disease shown below: patients with a past history of congestive heart failure, symptomatic coronary artery disease, arrhythmia uncontrolled by drugs, or myocardial infarction or angina pectoris requiring drug therapy; (4) patients whose ADRs due to pretreatment are not ameliorated to CTCAE Grade 1 (excluding haemoglobin, alopecia, and pigmentation); (5) patients who received other investigational drugs within 21 days before enrolment in this study; (6) patients who still have effects of surgery (including exploratory thoracotomy and thoracoscopy) performed within 28 days before enrolment in this study; (7) patients who received the following anticancer therapies before enrolment in this study: mitomycin C within 35 days before enrolment, bevacizumab within 28 days before enrolment, hormone therapy within 14 days before enrolment (except for use for medical castration in patients with prostate cancer), or other anticancer therapies (e.g., chemotherapy, molecular target drugs, biopharmaceuticals) within 21 days before enrolment; (8) patients who received radiation therapy within 21 days before enrolment in this study; (9) patients who received radiation therapy to 30% or more of haematopoietic bone marrow within the past 1 year; (10) patients with brain metastasis with clinical manifestations or brain metastasis requiring treatment; (11) patients with active infection requiring systemic treatment (e.g., with fever of 38°C or more due to infection); (12) patients with serious complications (e.g., intestinal paresis, ileus, interstitial pneumonia that can be seen on images, diabetes mellitus having poor glycaemic control even by drug therapy or severe diabetic complications, respiratory failure, renal failure, hepatic failure, psychiatric disorders, cerebrovascular accident, gastrointestinal ulceration requiring transfusion, wound or fracture in a healing process in which neovessels occur); (13) patients with accumulation of pleural fluid, ascitic fluid, or pericardial fluid requiring elimination of cavity fluid with a drain, or patients with pleural effusion for which treatment with a drain or by pleurodesis was performed within 21 days before enrolment; (14) patients with hypertension requiring treatment with two or more drugs (including combination products); (15) patients with positive test results for human immunodeficiency virus antibody, hepatitis B surface antigen, or hepatitis C virus (HCV) antibody within 14 days before enrolment (if the HCV antibody testing was positive, the patient can be enrolled when measurement of HCV-RNA showed a negative result); (16) pregnant women, lactating women, men and women who do not agree to use contraception during a specified period (men, 90 days; women, 180 days) after the last dose of the investigational drug. For women of childbearing potential, a pregnancy test should be performed before enrolment in this study to confirm a negative result; (17) patients with psychiatric disorders or psychiatric symptoms for whom it was considered difficult to participate in the study; (18) patients treated continuously with (oral or intravenous) systemic steroids; (19) patients who have received treatment with transfusion, blood component preparations, plasma fraction preparations, or G-CSF within 14 days before enrolment in this study; and (20) patients who were considered inappropriate for this study by the investigator or subinvestigator.

***Dose reduction criteria***

The dose of TAS-115 was reduced if a patient presented with any adverse drug reaction (ADR) that was considered a dose-limiting toxicity (DLT). In the case of an event that met the criteria for drug withdrawal or ADR that were likely to affect the treatment continuation, considerations to reduce the dosage were made by the investigator or subinvestigator. ADRs for which a dosage reduction should be considered were Grade ≥2 events, or continuing Grade 1 events consisting of anorexia, malaise, and nausea/vomiting. However, the dosage was not to be reduced during the DLT assessment period in Steps 1 and 2 unless DLT occurred. The dose was reduced by 25% using the dose at the time of the dose reduction decision as a base. Patients could undergo up to two dose reductions, and the minimum dosage was 100 mg/day. When the dose was reduced, a dose per administration was calculated, and the maximum value of a multiple of 50 mg (obtained by dropping all fractions of less than 50 mg/body), was used as the dose.

***Criteria for resumption of administration***

To resume TAS-115 treatment after discontinuation, the following criteria had to be met:

neutrophil count >1500/mm^3^, platelet count >75,000/mm^3^, nonhaematologic toxicity returned to Grade 2 or less, or adverse events (AEs) leading to the decision of drug withdrawal alleviated or resolved.

***Precautions and prohibited concomitant medications and therapies***

HP-β-CD, which is used as an excipient of TAS-115, may increase the plasma concentrations of orally administered concomitant medications. Therefore, administration of concomitant medications had to be avoided as much as possible for 4 hours before and after administration of TAS-115. Prohibited medications were other anticancer drugs (except for bisphosphonate preparations, denosumab, leuprorelin, goserelin, and degarelix used for medical castration in patients with prostate cancer) and other investigational drugs. Operative therapy, radiation therapy, thermotherapy, immunotherapy, hormone therapy, and antibody therapy (excluding radiation therapy for pain relief for bone metastasis) were prohibited during the study. Precautions were recommended for concomitant medications that metabolize cytochrome P450 (CYP) 2B6, CYP2C8, CYP2C9, CYP2C19, and CYP3A4/5 (except for drugs for external use); drugs and foods with inhibitory or inductive effects on CYP3A; and drugs and foods with inhibitory or inductive effects on P-gp.

**SUPPLEMENTARY RESULTS**

***Safety***

The most common adverse drug reactions occurring at an incidence of ≥30% were neutrophil count decreased (75%), aspartate aminotransferase increased (50%), and platelet count decreased (50%). The incidence of serious adverse events was 50% (*n*=10, 11 events), including one case each of retinal detachment, enterocolitis, gastrointestinal obstruction, pyrexia, peritonitis, sepsis, femoral neck fracture, acute myeloid leukaemia, pleural effusion, pharyngeal stenosis, and rash.
