# Supplementary material for: Efficacy and safety of TAS-115, a novel oral multi-kinase inhibitor, in osteosarcoma: an expansion cohort of a phase I study
Source: Invest New Drugs. 2021 Jun 12;39(6):1559–67. doi: 10.1007/s10637-021-01107-4 (PMC8541973; doi:10.1007/s10637-021-01107-4)
Supplement: Supplementary file 3 — (DOCX 56.5 kb) [file 10637_2021_1107_MOESM3_ESM.docx]

**SUPPLEMENTARY TABLES**

**Supplementary Table 1.** Serious adverse events, relationship with the study drug, and resulting study treatment adjustment

| **Preferred term** | **Relationship** | **Study treatment adjustment** |
| --- | --- | --- |
| Retinal detachment | Not related | Drug interrupted |
| Enterocolitis | Related | Drug interrupted |
| Gastrointestinal obstruction | Not related | None |
| Pyrexia | Related | Drug withdrawn,  drug interrupted,  dose reduced |
| Peritonitis | Not related | Drug interrupted |
| Sepsis | Not related | Drug withdrawn |
| Femoral neck fracture | Not related | None |
| Acute myeloid leukaemia | Not related | Drug withdrawn |
| Pleural effusion | Not related | Drug interrupted |
| Pharyngeal stenosis | Not related | None |
| Rash | Related | Drug withdrawn,  drug interrupted |

**Supplementary Table 2.** Treatment status of patients with osteosarcoma

| **Status** | ***N*=20**  **N (%)** |
| --- | --- |
| Dose reduction | 11 (55) |
| Reasons for dose reduction |  |
| ADR^a^ | 11 (55) |
| Drug interruption | 15 (75) |
| Reasons for drug interruption |  |
| ADR^b^ | 12 (60) |
| Other | 3 (15) |
| Ongoing | 4 (20) |
| Discontinued | 16 (80) |
| Reasons for discontinuation |  |
| Disease progression | 10 (50) |
| ADR^c^ | 3 (15) |
| Other | 3 (15) |
| Physician’s decision | 1 (5) |
| Non-drug related AE | 2 (10) |

ADR, adverse drug reaction; AE, adverse event.

^a^Neutrophil count decreased (*n*=6; 30%); platelet count decreased (*n*=4; 20%); pyrexia (*n*=2; 10%); febrile neutropenia, face oedema, oedema peripheral, hepatic function abnormal, hypersensitivity, aspartate aminotransferase increased, alanine aminotransferase increased, and rash (*n*=1 each; 5%).

^b^Neutrophil count decreased (*n*=5; 25%); platelet count decreased (*n*=4; 20%); face oedema, pyrexia, rash (*n*=2 each; 10%); febrile neutropenia, enterocolitis, nausea, vomiting, oedema peripheral, hypersensitivity, hypophosphataemia, and erythema (*n*=1 each; 5%).

^c^One case each of pyrexia, neutrophil count decreased, and rash.
